# Exploring the impact of oral health on the quality of life in older patients: a cross-sectional study

**DOI:** 10.1038/s41405-024-00244-1

**Published:** 2024-07-21

**Authors:** Nawaf H. Al Shammary

**Affiliations:** Department of Preventive Dentistry, College of Dentistry, University of Ha’il, P.O. Box 2440, Ha’il, 81481 Saudi Arabia

**Keywords:** Gerodontics, Dental epidemiology

## Abstract

**Objective:**

To investigate the significant impact of oral health on the quality of life of older individuals in Riyadh, Saudi Arabia, across various socioeconomic and demographic contexts.

**Methods:**

A cross- sectional study was conducted, involving the distribution of a translated online questionnaire based on the OHQoL-UK® tool to evaluate oral health-related quality of life OHRQoL. This included utilizing the Oral Health Quality of Life Scale to assess overall quality of life.

**Results:**

A total of 586 participants were involved in the study, with the majority being over 60 years old (77.1%). The mean score of OHRQoL was 3.79. The Social Dental Scale SDS had a mean score of 0.71. The General Oral Health Assessment GOHS scored 3.51 on average. The mean score of Dental Impact Profile DIP was 3.12. The Subjective Oral Health Status Indicators SOHSIs had a mean score of 3.82. The mean score of Oral Health Benefit of Life Inventory OHBLI averaged at 4.04, and Dental Impact on Daily Living DIDL scored an average of 4.05. The mean scores of OHRQoL and Oral Impacts on Daily Performance OIDP were 3.90 and 3.89 respectively. Cronbach’s Alpha values ranged from 0.854 to 0.939, with an overall questionnaire reliability of 0.977, indicating a good reliability of the study’s tool.

**Conclusion:**

Older adults exhibited lower OHRQoL compared to younger adults, particularly influenced by factors such as health insurance coverage, monthly income, and educational level. It is essential to develop health programs specifically tailored for senior adults to safeguard their overall health and quality of life. Making health and medical insurance obligatory and accessible to all individuals is crucial for enhancing their QoL and reducing the diseases.

## Introduction

Oral Health-Related Quality of Life (OHRQoL) refers to a multi-dimensional framework focusing on how oral health condition impact on an individual’s well-being, daily functioning, and overall quality of life [1]. The World Health Organization (WHO) further expands on this concept, defining health as not just the absence of disease but as a state of physical, mental and social-well-being [2].

The relationship between various oral health conditions and OHRQoL has been extensively investigated. The findings consistently showed a significant relationship between OHRQoL of older people and oral diseases, particularly periodontal disease [3]. Studies have also highlighted that the DMFT index has a negative impact on OHRQoL [3].

Poor oral health can lead to various functional, nutritional, esthetical, and psychological problems, ultimately affecting the overall quality of life [4]. Additionally, research has shown OHRQoL in the older person is influenced by clinical, demographic, and sociodemographic factors [3]. Moreover, the number of missing teeth and location of remaining teeth were identified as significant factors affecting quality of life [4].

Researchers have pointed out that experiencing pain and functional complaints are closely associated with impaired quality of life [4].

Therefore, it is crucial to adopt a model that focuses on various dimensions of oral health within a multi-dimensional framework [5, 6]. This study aimed to identify themes that embrace biopsychosocial dimensions of health including symptoms, physical abilities, emotional well-being and social communications to improve understanding about the effect of oral health and how it influences an individual’s overall well-being of [7, 8]. In addition, investigating Quality of Life (QoL) of individuals aged 65 and older is essential to understand their perspectives on oral health within the context of their cultural background, values living environment, expectations, concerns, and goals [9], [10].

Saudi Arabia is experiencing shifts in its demographic indices, characterized by an increase in life expectancy and a decrease in fertility rates. According to the United Nations Economic and Social Commission for Western Asia (2014), the median age in Saudi Arabia was 26.4 years, with males averaging 27.3 years for and females 25.3 years [11].

During this period, there has been a noticeable decline in fertility rates alongside a steady increase in life expectancy. Between 1980 and 1985, the fertility rate stood at 7.0 children per female, which dropped to 3.0 per female between 2005 and 2010 [12]. These shifts have caused the age pyramids to take on more slender shape over the past 30 years, raising concerns about the potential consequences of an aging population in the next decade.

This is evident through various indicators, such as the child-woman ratio, aged-child ratio, median age, and age dependency ratio [13]. This has garnered increased attention from global monitoring agencies, such as the WHO, United Nations, and U.S. Department of Commerce, revealing specific studies on demographic profiles of Arab countries. The mean life expectancy in 2014 was recorded as 74.8 years, with males living an average of 72.8 years and females 76.9 years. Furthermore, it is anticipated that life expectancy will continue to rise in the future [14, 15].

Despite this, research on the impact of oral health on the overall well-being of individuals aged 65 and above remains limited [12]. Therefore, this study aimed to investigate the significant implication of oral health on the quality of life among older people within various socioeconomic, and demographic contexts.

## Methodology

### Study design

A descriptive quantitative cross-sectional study was conducted, with a key strength being its capacity to allow for simultaneous comparisons of multiple factors. Online questionnaire was utilized to collect data over a period of 6 months from a sample comprising both adults and older individuals.

### Population and sample

#### Sampling

A random sampling approach was employed, utilizing the Richard Geiger equation. The study considered a margin of error of 5%, a confidence level of 95%, a population size, a response distribution of 50%, and a calculated sample size exceeding 550 expected responses. Following recommendations by the WHO, for minimal sample size, 586 participants were considered necessary [16].

The formula for sample size is presented,

Below is the formula used for the calculation.

$${{{{\rm{Unlimited}}}}}\,{{{{\rm{population}}}}} \!\! :\,{n}=\frac{{z}^{2}\times \hat{p}(1-\hat{p})}{{\varepsilon }^{2}}$$

$${{{{\rm{Finite}}}}}\,{{{{\rm{population}}}}} \!\! :\,{n^{\prime}} =\frac{n}{1+\frac{{z}^{2}\times \hat{p}(1-\hat{p})}{{\varepsilon }^{2}N}}$$

n =Sample size

z = Critical value = 1.960 with the selected confidence level of 95%

N = Population size

P = Sample proportion (that describes the number of people in a sample who have a certain trait or characteristic), ranging from 50 to 70% [17].

#### Data collection

The ethical approval was obtained from the hospitals involved in the study. Inclusion criteria comprised individuals aged 65 and over, who were resident of Saudi Arabia, and capable of participating in the survey independently. Exclusion criteria included individuals under 65 years old, not residing of Saudi Arabia, and those unable to participate independently. A questionnaire was used for data collection after introducing a study detail on its cover page.

The questionnaire was translated into Arabic to ensure reliability and suitability for the sample population. Demographic data including age, gender, education, marital status, insurance, household, insurance, income, as well as factors like functional limitations, social circle, self-confidence, communication, sleep, emotion, well-being and appearance were collected. Primary data collection was carried out through online surveys distributed via various social media. The questionnaire was estimated to require 30 min for completion, addressing all relevant questions.

During the development and evaluation of OHQoL-UK®, the tool comprised 8 subscales and 41 items [18, 19]. Participants rated each subscale on a five-point Likert scale, from “Strongly Disagree” to “Strongly Agree,” with scores of 1 for “Strongly Disagree,” 2 for “Disagree,” 3 for “Neutral,” 4 for “Agree,” and 5 for “Strongly Agree,”. However, the “SDS,” used a binary response format with “Yes” and “No” options. In this case, “Yes” was scored as 5, and “No” as 0 to ensure consistency with the other subscales.

The Likert scale is a valuable tool for measuring the level of enthusiasm. It is widely employed in surveys to understand the intensity of opinions of people on various issues [19]. To assess the opinions of survey takers, questions typically focus on their agreement or disagreement with specific statements or sets of questions. The validity of measurement scale was evaluated using three Likert scales to assess perspectives.

The study questions and hypothesis were formulated, and Statistical Package for Social Sciences, version 26, was utilized to analyze the collected data and test the research hypotheses. Frequencies and percentages were calculated to describe demographic variables. Cronbach’s Alpha reliability measures were determined to measure the strength of correlation and coherence between questionnaire items. Independent Sample T-test and one- way ANOVA were also calculated to perform comparisons.

## Results

### Internal consistency

The correlation coefficients between the scores of each domain within the SDS and the total score of the domain were statistically significant at a significance level of 0.01 (Table 1). All of these coefficients have positive values. This positive correlation suggests a robust internal consistency and a significant relationship between the domain and its items, thereby demonstrating the overall validity of the items in the first domain. Table 1 also indicates that the correlation coefficients between the scores of each item within the domain of General Oral Health Assessment GOHS and the total domain score were statistically significant at a significance level of 0.01. All of these coefficients have positive values, indicating a robust internal consistency and a significant relationship between the domain and its components. This demonstrates the overall validity of the items within the second domain.

**Table 1:** The internal consistency of all domains.

Item number	Pearson correlation	Item number	Pearson correlation
1. Top Management Commitment			
1	0.826^a^	3	0.831^a^
2	0.852^a^	4	0.826^a^
2. Customer Focus and Satisfaction			
1	0.834^a^	4	0.884^a^
2	0.825^a^	5	0.842^a^
3	0.865^a^		
3. Quality Policy			
1	0.768^a^	5	0.832^a^
2	0.772^a^	6	0.886^a^
3	0.822^a^	7	0.845^a^
4	0.834^a^		
4. Employee Training			
1	0.812^a^	4	0.894^a^
2	0.897^a^	5	0.880^a^
3	0.876^a^		
5. Employee Involvement			
1	0.862^a^	3	0.880^a^
2	0.900^a^	4	0.844^a^
6. Employee Empowerment			
1	0.824^a^	4	0.868^a^
2	0.890^a^	5	0.839^a^
3	0.812^a^	6	0.829^a^
7. Reward and Recognition			
1	0.903^a^	3	0.903^a^
2	0.926^a^		
8. Quality Policy			
1	0.816^a^	5	0.892^a^
2	0.845^a^	6	0.871^a^
3	0.862^a^	7	0.862^a^
4	0.846^a^		

^a^Correlation is significant at *α* = 0.01 or less.

The correlation coefficients between the scores of each item within the domain of Dental Impact profile DIP and the total domain score were statistically significant at a significance level of 0.01 (Table 1). Additionally, all of these coefficients have positive values, indicating a robust internal consistency and a significant relationship between the domain and its components. This demonstrates the overall validity of the items within the third domain.

Moreover, the correlation coefficients between the scores of each domain within the domain of Subjective Oral Health Status Indicators SOHSIs and the total domain score were statistically significant at a significance level of 0.01. Additionally, all of these coefficients have positive values, indicating a robust internal consistency and a significant relationship between the domain and its components. This demonstrates the overall validity of the items within the fourth domain. Table 1 indicates that the correlation coefficients between the scores of each item within the domain OHBLI and the total domain score were statistically significant at a significance level of 0.01. All of these coefficients have positive values, indicating a robust internal consistency and a relationship between the domain and its components. This demonstrates the overall validity of the items within the fifth domain. In addition, the correlation coefficients between the scores of each item within the domain of Dental Impact on Daily Living DIDL and the total domain score are statistically significant at a significance level of 0.01. All of these coefficients have positive values, indicating a robust internal consistency and a significant relationship between the domain and its items. This demonstrates the overall validity of the items within the sixth domain (Table 1). The results also suggest that the correlation coefficients between the scores of each item within the domain Oral health-related quality of life and the total domain score were statistically significant at a significance level of 0.01. All of these coefficients have positive values, indicating a robust internal consistency and a significant relationship between the domain and its items, demonstrating the overall validity of the items in the seventh domain.

The results shown in Table 1 suggest that the correlation coefficients between the scores of each item within the domain Oral Impacts on Daily Performance OIDP and the total domain score were statistically significant at a significance level of 0.01. Additionally, all of these coefficients have positive values, indicating a robust internal consistency and a significant relationship between the domain and its items, demonstrating the overall validity of the items in the eighth domain.

The results shown in Table 2 suggest that the correlation coefficients between the scores of each domain and the scale oral health are statistically significant at a significance level of 0.01. Additionally, all of these coefficients have positive values, indicating a robust internal consistency and a significant relationship between the scale and its domains, demonstrating the overall validity of the scale.

**Table 2:** The internal consistency of each domain with the total scale.

No	Domains	Pearson correlation
1	Social dental scale SDS	0.540
2	General Oral Health Assessment GOHS	0.866
3	Dental Impact Profile DIP	0.903
4	Subjective Oral Health Status Indicators SOHSIs	0.903
5	Oral Health Benefit of Life Inventory OHBLI	0.861
6	Dental Impact on Daily Living DIDL	0.860
7	Oral health- related quality of life OHRQoL	0.866
8	Oral Impacts on Daily Performance OIDP	0.875

**Table 3:** The Cronbach alpha results in this study.

No	Domains	No. items	Cronbach’s Alpha
1	Social Dental Scale SDS	4	0.854
2	General Oral Health Assessment GOHS	5	0.902
3	Dental Impact Profile DIP	7	0.920
4	Subjective Oral Health Status Indicators SOHSIs	5	0.921
5	Oral Health Benefit of Life Inventory OHBLI	4	0.893
6	Dental Impact on Daily Living DIDL	6	0.919
7	Oral health-related quality of life OHRQL	3	0.897
8	Oral Impacts on Daily Performance OIDP	7	0.939
	Total scale	41	0.977

Furthermore, the findings presented in Table 3 indicated that the Cronbach’s Alpha values ranged from 0.854 to 0.939, with an overall questionnaire reliability of 0.977. These findings indicate that the questionnaire exhibited good reliability.

### Demographic characteristics

The study included 586 participants, the majority of whom (77.1%) were over 60 years. About 13.4% fell between the ages of 50 and 60 years old, and 9.5% were under 50 years old. The gender distribution was approximately equal, with 49.7% male and 50.3% female participants.

Regarding education, the majority (41.6%) had a university education, followed by 23.2% with primary education, 24.2% with a high school education, and 11% with non-university higher education. In terms of marital status, the majority (72.1%) were married, 12.3% were widowed, 7% were divorced, 2.5% were separated, and 6.1% were single. When it comes to health insurance, 61.7% had it, while 38.3% did not have any health insurance

Regarding income, the majority (26.6%) had an income between 5000 and 10,000 SR, 25.4% had an income between 10,001 and 20,000 SR, 16.9% had an income of less than 5000 SR, 8% had an income between 20,001 and 30,000 SR, and 4.9% had an income of more than 30,000 SR. Table 4 presents a detailed overview of demographic data.

**Table 4:** Demographic characteristics.

Variables	Categories	*N* (%)
Gender	Male	292 (49.7)
	Female	294 (50.3)
Education	Primary	136 (23.2)
	High school	142 (24.2)
	Non-university higher education	64 (11)
	University	244 (41.6)
Marital status	Single	35 (6.1)
	Married	422 (72.1)
	Divorced	41 (7)
	Separated	15 (2.5)
	Widowed	73 (12.3)
Health Insurance	Yes	362 (61.7)
	No	224 (38.3)
Income	Less than 5000 SR	99 (16.9)
	From 5000–9900 SR	156 (26.6)
	From 10,000–19,900 SR	149 (25.4)
	From 20,000–30,000 SR	47 (8)
	More than 30,000 SR	29 (4.9)
	Reject to announce	106 (18.2)

### Results of OHRQoL

The results showed that the mean score for OHRQoL was 3.79. The mean score of the SDS was 0.71. The mean score of GOHS was 3.51. The mean score of DIP was 3.12. The mean score of SOHSIs was 3.82. The mean score of OHBLI was 4.04, and for DIDL was 4.05. The mean score of Oral health-related quality of life was 3.90, and for OIDP was 3.89. Findings related to participants’ responses about OHRQoL in senior adults are presented in Table 5.

**Table 5:** Responses of the participants about OHRQoL in senior adults.

No	Items	Mean	Standard Deviation	Median	Interquartile range
1	SDS	3.42	1.94	5	2.5
2	General Oral Health Assessment GOHS	3.51	0.89	4.0	1.1
3	Dental Impact Profile DIP	3.12	1.11	4.0	1.0
4	Subjective Oral Health Status Indicators SOHSIs	3.82	0.91	4.0	1.0
5	Oral Health Benefit of Life Inventory OHBLI	4.04	0.85	4.0	1.0
6	Dental Impact on Daily Living DIDL	4.05	0.80	4.2	1.0
7	Oral health-related quality of life OHRQoL	3.90	0.92	4.0	1.3
8	Oral Impacts on Daily Performance OIDP	3.86	0.84	4.0	1.0
	Total scale	3.86	0.80	4.07	0.9

The results showed that the level of the SDS increases by 0.134 when individuals aged more than 60 years change to the 50–60 age range (*β* = −0.125, *P* < 0.001). This level increases by 0.287 when the education level changes from university to illiterate (*β* = 0.287, *P* < 0.001), by 0.283 when the education level changes from university to primary (*β* = 0.283*, P* < 0.001), and by 0.168 when the education level changes from university to high school (*β* = 0.168, *P* = 0.002). Furthermore, a 0.092 increase occurs when changing from a retired to full-time employment. The GOHS level increases by 0.312 when transitioning from University to primary education level (*β* = 0.312, *P* = 0.003), by 0.233 when income changes from 10,000–19,900 SR to 5000–9900 SR (*β* = 0.233, *P* = 0.001), and increases by 0.334 when income changes from 10,000–19,900 to 20,000–30,000 SR (β = 0.334, *P* = 0.002). The DIP level increases by 0.273 when the education level changes from university to primary (*β* = 0.273, *P* = 0.001) and by 0.259 when the income changes from 10,000–19,900 to 5000–9900 SR (*β* = 0.259, *P* = 0.016). The SOHSIs level increases by 0.185 for participants with health insurance (*β* = 0.185, *P* = 0.03) and by 0.290 when transition from university to primary education level (*β* = 0.290, *P* = 0.001). The OHBLI level decreases by 0.381 when income changes from 10,000–19,900 to more than 30000 SR (*β* = –0.381, *P* = 0.003). The OHRQoL level increases by 0.591 when transition from university to illiterate education level (*β* = 0.591, *P* = 0.001), by 0.366 when moving from university to primary education level (*β* = 0.366, *P* = 0.002), and by 0.285 when moving from university to high school education level (*β* = 0.285, *P* < 0.001). The OIDP level increases by 0.443 when shifting from university to illiterate education level (*β* = 0.443, *P* = 0.001), by 0.410 when transition from university to primary education level (*β* = 0.410, *P* < 0.001). The OIDP increases by 0.296 when moving from university to high school education level (*β* = 0.296, *P* < 0.001), and by 0.275 when moving from university to high school education level (*β* = 0.275, *P* = 0.001).

## Discussion

The importance of maintaining good oral health is frequently overlooked by the older person when considering health problems like cardiovascular disease, neurological disease, diabetes, and cancer that severely affect their quality of life [18].

Numerous studies have addressed the need to prioritize oral health in older individuals and adults [18], especially those dealing with long-term health conditions that can impact their oral well-being [19].

This study aimed to evaluate the oral health status and quality of life of adults in Riyadh, Saudi Arabia among 586 participants, predominantly over 60 years old.

Gender equality in participation was ensured to avoid bias, as previously recommended [20]. A significant proportion of participants lacked health insurance, mostly due to moderate monthly income, potentially leading to neglect of oral health due to financial constraints. Previous research have shown a lower OHRQoL in individuals from social groups with low monthly incomes and limited educational attainment [21]. Additionally, researchers have observed reduced dental care utilization among older people, especially within low-income populations. This trend is linked to their inability to perceive the need to visit the dentist, fear, anxiety, past negative experience and insufficient awareness of dental problems [22].

The findings revealed a moderate level of OHRQoL, with a mean score of 3.79. This moderate quality of life was associated with various negative impacts on oral health, as reported by Bastos et al. [23] study. Researchers found a significant association between moderate to severe frailty and OHRQoL among community-dwelling older individuals in the community [23]. Moreover, the findings identified several factors influencing the social dental scale. Specifically, the SDS increased by 0.134 when individuals aged over 60 moved to the 50–60 age group. Furthermore, the scale increased by 0.287 when moving from university education level to illiteracy, by 0.283 when the education level changes from university to primary education, and by 0.168 when shifting to high school education. Furthermore, shifting from a retired to full-time occupation led to an increase of 0.092 in the scale.

Older people experience various dental pathologies including periodontal disease, dental caries, missing teeth, oral mucosal lesions, oral infections, and temporomandibular pathology. These problems may result in a reduced intake of healthy food such as fruits and vegetables while increasing the consumption of soft foods high in saturated fats and cholesterol [18].

Previous study undertaken by Raphael (2017) reported the increased risk of periodontal diseases among older adults, increased risk of root cavities, and bad general oral health [24].

Researchers also reported the frequency of xerostomia, which can facilitate the proliferation of oral pathogens, leading to dental caries. Other clinical features have been documented in studies, commonly seen in the older person, including candidiasis, burning tongue, tooth surface loss, fissuring of the tongue, difficulty with swallowing and speech, mucositis, and loss of taste perception [10].

Moreover, the prevalence of oral cancer was found to be higher among individuals over 45 years, with a twofold higher incidence observed in males compared to females.

Dental problems can also lead to reduction in social interaction which causes psychosocial distress [18].

The present study underscores the importance of addressing oral health challenges among older individuals to improve their overall well-being and quality of life.

In addition, the GOHS and the DIP levels increase by 0.312 and 0.273 respectively, when the education level changes from university to primary education (*P* < 0.001). They also increase by 0.233 and 0.259 when the income changes from 10,000–19900 to 5000–9900 SR (*β* = 0.233, *P* < 0.001). This contrasts the findings of Márquez-Arrico et al. [25], which indicated that 41.5% of participants had a low level of understanding, while 58.5% exhibited a high level of knowledge [25]. A significant relationship was found between educational attainment and oral health knowledge. There is a correlation between oral hygiene behaviors and a better understanding of oral health. Specifically, the use of dental floss, a higher number of teeth, and a lower prevalence of partial prosthesis are associated with increased levels of oral health awareness. There is also a notable correlation between the quality of life related to oral health and individual’s level of knowledge regarding oral health.

Furthermore, there is a relationship between an individual’s level of education and their understanding of oral health. However, having knowledge about oral health does not always lead to the adoption of proper oral hygiene practices. This is consistent with Firmino et al. [26], study, who reported that individuals who responded “sometimes”, “often”, or “very often” to at least one item as belonging to the case group, indicating a negative impact on OHRQoL [26].

It is essential to establish effective preventive strategies and individualized treatment protocols among older patients. These individuals require diet education, hygiene education, and patient-specific measures to improve their healthcare [10].

Several methods have been suggested to improve the dental care for the older person. One method involves public health campaigns to raise awareness about oral health. This can involve assembling a team of professionals to implement dental programs. Additionally, implementing new models such as mobile technologies, tele-dentistry, adoption of oral health teams, and integrating geriatric and primary care services are suggested ways to improve oral health in the older person [27].

Moreover, educating caregivers about dental hygiene, oral health promotion, the use of fluorides, denture care, and regular dental check-ups has proven highly beneficial in improving oral health of older people [28].

Cases and controls were carefully selected to ensure comparability in terms of age, sex, and monthly household income. The categorization of monthly household income was based on the monthly minimum wage in Brazil.

## Conclusion

The results of this study showed that the OHRQoL was moderately low. There was a significant difference in the scale between participant due to education level, health insurance, and income. Across various dimensions, the scores for SDS, GOHS, DIP, SOHSIs, the OHBLI, the DIDL, the OHRQoL, and the OIDP were relatively high. Age and education had a significant effect on the SDS. Education and income had a significant effect on the GOHS. Education and income had a significant effect on the DIP. Health insurance and education had a significant effect on the SOHSIs. Income had a significant effect on the OHBLI. Education had a significant effect on the OHRQoL. Education had a significant effect on the OIDP. It is essential to enhance health programs specifically tailored for senior adults to safeguard their overall health and quality of life. Making health and medical insurance obligatory and accessible to all individuals is crucial for enhancing their quality of life and reducing the prevalence of diseases.

## Data Availability

All data is available upon request when needed.
